# Supplementary material for: Population-based trends in hospitalizations due to injection drug use-related serious bacterial infections, Oregon, 2008 to 2018
Source: PLoS One. 2020 Nov 9;15(11):e0242165. doi: 10.1371/journal.pone.0242165 (PMC7652306; doi:10.1371/journal.pone.0242165)
Supplement: S1 Data — (DOCX) [file pone.0242165.s001.docx]

**ICD-9 and ICD-10 code equivalents for Hospital Discharge Data: Injection drug use related hospitalizations**

Source: http://www.icd9data.com/2015/Volume1/default.htm;

The definition of an IDU is if the diagnosis codes for a patient-visit (as opposed to across visits) contain at least one drug use code and one infection code

At least one of the drug codes for: opiates, cocaine, amphetamines, sedatives, or other drugs

and

At least one of the infection codes for: endocarditis, bacteremia\sepsis, osteomyelitis, or skin\soft tissue infections

**Opiates ICD-9s**

304.00 Opioid type dependence, unspecified

304.01 Opioid type dependence, continuous

304.02 Opioid type dependence, episodic

304.70 Combinations of opioid type drug with any other drug dependence, unspecified

304.71 Combinations of opioid type drug with any other drug dependence, continuous

304.72 Combinations of opioid type drug with any other drug dependence, episodic

305.50 Opioid abuse, unspecified

305.51 Opioid abuse, continuous

305.52 Opioid abuse, episodic

**Opiates ICD-10s**

F11.10 Opioid abuse, uncomplicated

F11.121 …… delirium

F11.122 …… with perceptual disturbance

F11.129 …… unspecified

F11.14 Opioid abuse with opioid-induced mood disorder

F11.150 Opioid abuse with opioid-induced psychotic disorder, delusions

F11.151 …… with hallucinations

F11.159 …… unspecified

F11.181 Opioid abuse with opioid-induced sexual dysfunction

F11.182 Opioid abuse with opioid-induced sleep disorder

F11.188 Opioid abuse with other opioid-induced disorder

F11.19 Opioid abuse with unspecified opioid-induced disorder

F11.20 Opioid dependence, uncomplicated

F11.220 Opioid dependence with intoxication, uncomplicated

F11.221 …. delirium

F11.222 …… with perceptual disturbance

F11.229 …… unspecified

F11.23 Opioid dependence with withdrawal

F11.24 Opioid dependence with opioid-induced mood disorder

F11.250 Opioid dependence with opioid-induced psychotic disorder, delusions

F11.251 …… with hallucinations

F11.259 …… unspecified

F11.281 Opioid dependence with opioid-induced sexual dysfunction

F11.282 Opioid dependence with opioid-induced sleep disorder

F11.288 Opioid dependence with other opioid-induced disorder

F11.29 Opioid Dependence with unspecified opioid-induced disorder

F11.90 Opioid use, uncomplicated

F11.920 Opioid use with intoxication, uncomplicated

F11.921 …… delirium

F11.922 …… with perceptual disturbance

F11.929 …… unspecified

F11.93 Opioid use, with withdrawal

F11.94 Opioid use, with opioid-induced mood disorder

F11.950 Opioid use, with delusions

F11.951 …… with hallucinations

F11.959 …… unspecified

F11.981 Opioid use, unspecified with opioid-induced sexual dysfunction

F11.982 Opioid use, unspecified with opioid-induced sleep disorder

F11.988 Opioid use, unspecified with other opioid-induced disorder

F11.99 Opioid use with unspecified opioid-induced disorder

**Cocaine ICD-9s**

304.20 Cocaine dependence, unspecified

304.21 Cocaine dependence, continuous

304.22 Cocaine dependence, episodic

305.60 Cocaine abuse, unspecified

305.61 Cocaine abuse, continuous

305.62 Cocaine abuse, episodic

**Cocaine ICD-10s**

F14.10 Cocaine abuse, uncomplicated

F14.120 Cocaine abuse with intoxication, uncomplicated

F14.121 …… with delirium

F14.122 …… with perceptual disturbance

F14.129 …… unspecified

F14.14 …… with cocaine-induced mood disorder

F14.150 Cocaine abuse with cocaine-induced psychotic disorder, with delusions

F14.180 Cocaine abuse with other cocaine-induced anxiety disorder

F14.181 Cocaine abuse with cocaine-induced sexual dysfunction

F14.182 Cocaine abuse with cocaine-induced sleep disorder

F14.188 Cocaine abuse with other cocaine-induced disorder

F14.19 …… with unspecified cocaine-induced disorder

F14.20 Cocaine dependence, uncomplicated

F14.220 Cocaine dependence with intoxication, uncomplicated

F14.221 …… delirium

F14.222 …… with perceptual disturbance

F14.229 …… unspecified

F14.23 …… with withdrawal

F14.24 …… with cocaine-induced mood disorder

F14.250 Cocaine dependence with cocaine-induced psychotic disorder, with delusions

F14.251 …… with hallucinations

F14.259 …… unspecified

F14.280 Cocaine dependence with cocaine-induced anxiety disorder

F14.281 Cocaine dependence with cocaine-induced sexual dysfunction

F14.282 Cocaine dependence with cocaine-induced sleep disorder

F14.288 Cocaine dependence with other cocaine-induced disorder

F14.29 …… with unspecified cocaine-induced disorder

F14.90 Cocaine use, unspecified, uncomplicated

F14.920 Cocaine use, unspecified with intoxication, uncomplicated

F14.921 …… delirium

F14.922 …… with perceptual disturbance

F14.929 …… unspecified

F14.94 …… with cocaine-induced mood disorder

F14.950 Cocaine use, unspecified with cocaine-induced psychotic disorder, with delusions

F14.951 …… with hallucinations

F14.959 …… unspecified

F14.980 Cocaine use, unspecified with cocaine-induced anxiety disorder

F14.981 Cocaine use, unspecified with cocaine-induced sexual dysfunction

F14.982 Cocaine use, unspecified with cocaine-induced sleep disorder

F14.988 Cocaine use, unspecified with other cocaine-induced disorder

F14.99 …… with unspecified cocaine-induced disorder

**Amphetamines ICD-9s**

304.40 Amphetamine and other psychostimulant dependence, unspecified

304.41 Amphetamine and other psychostimulant dependence, continuous

304.42 Amphetamine and other psychostimulant dependence, episodic

305.70 Amphetamine or related acting sympathomimetic abuse, unspecified

305.71 Amphetamine or related acting sympathomimetic abuse, continuous

305.72 Amphetamine or related acting sympathomimetic abuse, episodic

**Amphetamines ICD-10s**

F15.10 Other stimulant abuse, uncomplicated

F15.120 Other stimulant abuse with intoxication, uncomplicated

F15.121 …… delirium

F15.122 …… with perceptual disturbance

F15.129 …… unspecified

F15.14 …… with stimulant-induced mood disorder

F15.150 Other stimulant abuse with stimulant-induced psychotic disorder with delusions

F15.151 …… with hallucinations

F15.159 …… unspecified

F15.180 Other stimulant abuse with stimulant-induced anxiety disorder

F15.181 Other stimulant abuse with stimulant-induced sexual dysfunction

F15.182 Other stimulant abuse with stimulant-induced sleep disorder

F15.188 Other stimulant abuse with other stimulant-induced disorder

F15.19 …… with unspecified stimulant-induced disorder

F15.20 Other stimulant dependence, uncomplicated

F15.220 Other stimulant dependence with intoxication, uncomplicated

F15.221 …… delirium

F15.222 …… with perceptual disturbance

F15.229 …… unspecified

F15.23 …… with withdrawal

F15.24 …… with stimulant-induced mood disorder

F15.250 Other stimulant dependence with stimulant-induced psychotic disorder with delusions

F15.251 …… with hallucinations

F15.259 …… unspecified

F15.280 Other stimulant dependence with stimulant-induced anxiety disorder

F15.281 Other stimulant dependence with stimulant-induced sexual dysfunction

F15.282 Other stimulant dependence with stimulant-induced sleep disorder

F15.288 Other stimulant dependence with other stimulant-induced disorder

F15.29 …… with unspecified stimulant-induced disorder

F15.90 Other stimulant use, unspecified, uncomplicated

F15.920 Other stimulant use, unspecified with intoxication, uncomplicated

F15.921 …… delirium

F15.922 …… with perceptual disturbance

F15.929 …… unspecified

F15.93 …… with withdrawal

F15.94 …… with stimulant-induced mood disorder

F15.950 Other stimulant use, unspecified with stimulant-induced psychotic disorder, with delusions

F15.951 …… with hallucinations

F15.959 …… unspecified

F15.980 Other stimulant use, unspecified with stimulant-induced anxiety disorder

F15.981 Other stimulant use, unspecified with stimulant-induced sexual dysfunction

F15.982 Other stimulant use, unspecified with stimulant-induced sleep disorder

F15.988 Other stimulant use, unspecified with other stimulant-induced disorder

F15.99 …… with unspecified stimulant-induced disorder

**Sedatives ICD-9s**

304.10 Sedative, hypnotic or anxiolytic dependence, unspecified

Specific code 304.11 Sedative, hypnotic or anxiolytic dependence, continuous

Specific code 304.12 Sedative, hypnotic or anxiolytic dependence, episodic

305.40 Sedative, hypnotic or anxiolytic abuse, unspecified

305.41 Sedative, hypnotic or anxiolytic abuse, continuous

305.42 Sedative, hypnotic or anxiolytic abuse, episodic

**Sedatives ICD-10s**

F13 Sedative, hypnotic, or anxiolytic related disorders

F13.1 Sedative, hypnotic or anxiolytic-related abuse

F13.2 Sedative, hypnotic or anxiolytic-related dependence

F13.9 Sedative, hypnotic or anxiolytic-related use, unspecified

**Other injected drugs ICD-9s**

304.80 Combinations of drug dependence excluding opioid type drug, unspecified

304.81 Combinations of drug dependence excluding opioid type drug, continuous

304.82 Combinations of drug dependence excluding opioid type drug, episodic

304.90 Unspecified drug dependence, unspecified

304.91 Unspecified drug dependence, continuous

304.92 Unspecified drug dependence, episodic

305.90 Other, mixed, or unspecified drug abuse, unspecified

305.91 Other, mixed, or unspecified drug abuse, continuous

305.92 Other, mixed, or unspecified drug abuse, episodic

304.60 Other specified drug dependence, unspecified

304.61 Other specified drug dependence, continuous

304.62 Other specified drug dependence, episodic

**Other injected drugs ICD-10s**

F19.10 Other psychoactive substance abuse, uncomplicated

F19.120 Other psychoactive substance abuse with intoxication, uncomplicated

F19.121 …… delirium

F19.122 …… with perceptual disturbances

F19.129 …… unspecified

F19.14 …… with psychoactive substance-induced mood disorder

F19.150 Other psychoactive substance abuse with psychoactive substance-induced psychotic disorder, with delusions

F19.151 …… with hallucinations

F19.159 …… unspecified

F19.16 …… with psychoactive substance-induced persisting amnestic disorder

F19.17 …… with psychoactive substance-induced persisting dementia

F19.180 Other psychoactive substance abuse with psychoactive substance-induced anxiety disorder

F19.181 Other psychoactive substance abuse with psychoactive substance-induced sexual dysfunction

F19.182 Other psychoactive substance abuse with psychoactive substance-induced sleep disorder

F19.188 Other psychoactive substance abuse with other psychoactive substance-induced disorder

F19.19 …… with unspecified psychoactive substance-induced disorder

F19.20 Other psychoactive substance dependence, uncomplicated

F19.21 …… in remission

F19.220 Other psychoactive substance dependence with intoxication, uncomplicated

F19.221 …… delirium

F19.222 …… with perceptual disturbance

F19.229 …… unspecified

F19.230 Other psychoactive substance dependence with withdrawal, uncomplicated

F19.231 …… delirium

F19.232 …… with perceptual disturbance

F19.239 …… unspecified

F19.24 …… with psychoactive substance-induced mood disorder

F19.250 Other psychoactive substance dependence with psychoactive substance-induced psychotic disorder, with delusions

F19.251 …… with hallucinations

F19.259 …… unspecified

F19.26 …… with psychoactive substance-induced persisting amnestic disorder

F19.27 …… with psychoactive substance-induced persisting dementia

F19.280 Other psychoactive substance dependence with psychoactive substance-induced anxiety disorder

F19.281 Other psychoactive substance dependence with psychoactive substance-induced sexual dysfunction

F19.282 Other psychoactive substance dependence with psychoactive substance-induced sleep disorder

F19.288 Other psychoactive substance dependence with other psychoactive substance-induced disorder

F19.29 …… with unspecified psychoactive substance-induced disorder

F19.90 Other psychoactive substance use, unspecified, uncomplicated

F19.920 Other psychoactive substance use, unspecified with intoxication, uncomplicated

F19.921 …… with delirium

F19.922 …… with perceptual disturbance

F19.929 …… unspecified

F19.930 Other psychoactive substance use, unspecified with withdrawal, uncomplicated

F19.931 …… delirium

F19.932 …… with perceptual disturbance

F19.939 …… unspecified

F19.94 …… with psychoactive substance-induced mood disorder

F19.950 Other psychoactive substance use, unspecified with psychoactive substance-induced psychotic disorder, with delusions

F19.951 …… with hallucinations

F19.959 …… unspecified

F19.96 …… with psychoactive substance-induced persisting amnestic disorder

F19.97 …… with psychoactive substance-induced persisting dementia

F19.980 Other psychoactive substance use, unspecified with psychoactive substance-induced anxiety disorder

F19.981 Other psychoactive substance use, unspecified with psychoactive substance-induced sexual dysfunction

F19.982 Other psychoactive substance use, unspecified with psychoactive substance-induced sleep disorder

F19.988 Other psychoactive substance use, unspecified with other psychoactive substance-induced disorder

F19.99 …… with unspecified psychoactive substance-induced disorder

**Endocarditis ICD-9s**

112.81 Candidal endocarditis

421.0 Acute and subacute bacterial endocarditis

421.1 Acute and subacute infective endocarditis in diseases classified elsewhere

421.9 Acute endocarditis, unspecified

424.90 Endocarditis, valve unspecified, unspecified cause

424.91 Endocarditis in diseases classified elsewhere

424.99 Other endocarditis, valve unspecified

424.0 Mitral valve disorders

424.1 Aortic valve disorders

424.2 Tricuspid valve disorders, specified as nonrheumatic

424.3 Pulmonary valve disorders

**Endocarditis ICD-10s**

B37.6 Candidal endocarditis

I33 Acute and subacute endocarditis

I34 Nonrheumatic mitral valve disorders

I35 Nonrheumatic aortic valve disorders

I36 Nonrheumatic tricuspid valve disorders

I37 Nonrheumatic pulmonary valve disorders

I38 Endocarditis, valve unspecified

I39 Endocarditis and heart valve disorders in diseases classified elsewhere

**Bacteremia\Sepsis ICD-9s**

038.0 Streptococcal septicemia

038.19 Other staphylococcal septicemia

038.2 Pneumococcal septicemia [Streptococcus pneumoniae septicemia]

038.3 Septicemia due to anaerobes

038.49 Other septicemia due to gram-negative organisms

038.8 Other specified septicemias

038.9 Unspecified septicemia

415.12 Septic pulmonary embolism

422.91 Idiopathic myocarditis

422.92 Septic myocarditis

038.10 Staphylococcal septicemia, unspecified

038.11 Methicillin susceptible Staphylococcus aureus septicemia

038.12 Methicillin resistant Staphylococcus aureus septicemia

038.40 Septicemia due to gram-negative organism, unspecified

038.41 Septicemia due to hemophilus influenzae [H. influenzae]

038.42 Septicemia due to escherichia coli [E. coli]

038.43 Septicemia due to pseudomonas

038.44 Septicemia due to serratia

449 Septic arterial embolism

785.52 Septic shock

790.7 Bacteremia

995.90 Systemic inflammatory response syndrome, unspecified

995.91 Sepsis

995.92 Severe sepsis

995.93 Systemic inflammatory response syndrome\without acute organ dysfunction

**Bacteremia\Sepsis ICD-10s**

A40 Streptococcal sepsis

A41 Other sepsis

I26.90 Septic pulmonary embolism without acute cor pulmonale

I40.0 Infective myocarditis

I76 Septic arterial embolism

R65.21 Severe sepsis with septic shock

R78.81 Bacteremia

R65.10 Systemic inflammatory response syndrome without acute organ dysfunction

R65.20 Severe sepsis without septic shock

A41.9 Sepsis, unspecified organism

**Osteomyelitis ICD-9s**

730.0 Acute osteomyelitis

730.1 Chronic osteomyelitis

730.2 Unspecified osteomyelitis

730.9 Unspecified infection of bone

**Osteomyelitis ICD-10s**

M86.1 Other acute osteomyelitis

M86.2 Subacute osteomyelitis

M86.9 Osteomyelitis, unspecified

M46.2 Osteomyelitis of vertebra

M86.9 Osteomyelitis, unspecified

M46.3 Infection of intervertebral disc (pyogenic)

**Skin\Soft tissue disease ICD-9s**

040.0 Gas gangrene

324.0 Intracranial abscess

324.1 Intraspinal abscess

324.9 Intracranial and intraspinal abscess of unspecified site

326 Late effects of intracranial abscess or pyogenic infection

567.31 Psoas muscle abscess

681.0 Cellulitis and abscess of finger

681.00 Cellulitis and abscess of finger, unspecified

681.01 Felon

681.02 Onychia and paronychia of finger

681.1 Cellulitis and abscess of toe

681.10 Cellulitis and abscess of toe, unspecified

681.11 Onychia and paronychia of toe

681.9 Cellulitis and abscess of unspecified digit

682.0 Cellulitis and abscess of face

682.1 Cellulitis and abscess of neck

682.2 Cellulitis and abscess of trunk

682.3 Cellulitis and abscess of upper arm and forearm

682.4 Cellulitis and abscess of hand, except fingers and thumb

682.5 Cellulitis and abscess of buttock

682.6 Cellulitis and abscess of leg, except foot

682.7 Cellulitis and abscess of foot, except toes

682.8 Cellulitis and abscess of other specified sites

682.9 Cellulitis and abscess of unspecified sites

686.9 Unspecified local infection of skin and subcutaneous tissue

728.86 Necrotizing fasciitis

723.6 Panniculitis specified as affecting neck

729.30 Panniculitis, unspecified site

729.39 Panniculitis, other site

785.4 Gangrene

**Skin\Soft tissue disease ICD-10s**

A48.0 Gas gangrene

G06 Intracranial and intraspinal abscess and granuloma

G09 Sequelae of inflammatory diseases of central nervous system

K68.12 Psoas muscle abscess

L03 Cellulitis and acute lymphangitis

L08.9 Local infection of the skin and subcutaneous tissue, unspecified

L98.9 Disorder of the skin and subcutaneous tissue, unspecified

M72.6 Necrotizing fasciitis

M54.02 Panniculitis affecting regions of neck and back, cervical region

M79.3 Panniculitis, unspecified

I96 Gangrene, not elsewhere classified

-=-=-=-=-=-=-=-=-=-=-=-=-=-=-=-=-=-=-=-=-=-=-

**Important exclusions**

**Remission suggests non-active IDU (ICD-9)**

304.03 Opioid type dependence, in remission

304.73 Combinations of opioid type drug with any other drug dependence, in remission

305.53 Opioid abuse, in remission

304.63 Other specified drug dependence, in remission

**Poisoning codes are not exclusive to IDU, but may be oral, rectal, smoked**

E850.0 Accidental poisoning by heroin

E850.2 Accidental poisoning by other opiates and related narcotics

E950.4 Suicide and self-inflicted poisoning by other specified drugs and medicinal substances

E980.4 Poisoning by other specified drugs and medicinal substances, undetermined whether accidentally or purposely inflicted

965.00 Poisoning by opium (alkaloids), unspecified

E962.0 Assault by drugs and medicinal substances

E980.0 Poisoning by analgesics, antipyretics, and antirheumatics, undetermined whether accidentally or purposely inflicted

965.09 Poisoning by other opiates and related narcotics

965.01 Poisoning by heroin

965.09 Poisoning by other opiates and related narcotics

Not specific to the kind of drug (ICD-9 used with drug codes)

292.0 Drug withdrawal

292.11 Drug-induced psychotic disorder with delusions

292.12 Drug-induced psychotic disorder with hallucinations

292.2 Pathological drug intoxication

292.81 Drug-induced delirium

292.84 Drug-induced mood disorder

292.85 Drug induced sleep disorders

292.89 Other specified drug-induced mental disorders

292.9 Unspecified drug-induced mental disorder

**Remission suggests non-active IDU (ICD-10)**

F11.21 Opioid dependence, in remission

F19.21 Other psychoactive substance dependence, in remission

**Poisoning codes are not exclusive to IDU, but may be oral, rectal, smoked (ICD-10)**

T40.0X1A Poisoning by opium, accidental (unintentional), initial encounter

T40.0X2A Poisoning by opium, intentional self-harm, initial encounter

T40.0X3A Poisoning by opium, assault, initial encounter

T40.0X4A Poisoning by opium, undetermined, initial encounter

T40.2X1A Poisoning by other opioids, accidental (unintentional), initial encounter

T40.2X2A Poisoning by other opioids, intentional self-harm, initial encounter

T40.2X3A Poisoning by other opioids, assault, initial encounter

T40.2X4A Poisoning by other opioids, undetermined, initial encounter

T40.4X1A Poisoning by other synthetic narcotics, accidental (unintentional), initial encounter

T40.4X2A Poisoning by other synthetic narcotics, intentional self-harm, initial encounter

T40.4X3A Poisoning by other synthetic narcotics, assault, initial encounter

T40.4X4A Poisoning by other synthetic narcotics, undetermined, initial encounter

T40.601A Poisoning by unspecified narcotics, accidental (unintentional), initial encounter

T40.602A Poisoning by unspecified narcotics, intentional self-harm, initial encounter

T40.603A Poisoning by unspecified narcotics, assault, initial encounter

T40.604A Poisoning by unspecified narcotics, undetermined, initial encounter

T40.691A Poisoning by other narcotics, accidental (unintentional), initial encounter

T40.692A Poisoning by other narcotics, intentional self-harm, initial encounter

T40.693A Poisoning by other narcotics, assault, initial encounter

T40.694A Poisoning by other narcotics, undetermined, initial encounter

**Remission or codes not specific to IDU (Use may be oral, rectal, or smoked)**

304.23 Cocaine dependence, in remission

970.81 Poisoning by cocaine

E854.2 Accidental poisoning by psychostimulants

E950.4 Suicide and self-inflicted poisoning by other specified drugs and medicinal substances

E962.0 Assault by drugs and medicinal substances

E980.4 Poisoning by other specified drugs and medicinal substances, undetermined whether accidentally or purposely inflicted

**Remission or codes not specific to IDU (Use may be oral, rectal, or smoked)**

F14.21 Cocaine dependence, in remission

T40.5X1A Poisoning by cocaine, accidental (unintentional), initial encounter

T40.5X2A Poisoning by cocaine, intentional self-harm, initial encounter

T40.5X3A Poisoning by cocaine, assault, initial encounter

T40.5X4A Poisoning by cocaine, undetermined, initial encounter

**Remission or codes not specific to IDU (Use may be oral, rectal, or smoked)**

304.43 Amphetamine and other psychostimulant dependence, in remission

305.73 Amphetamine or related acting sympathomimetic abuse, in remission

969.72 Poisoning by amphetamines

E854.2 Accidental poisoning by psychostimulants

E950.3 Suicide and self-inflicted poisoning by tranquilizers and other psychotropic agents

E962.0 Assault by drugs and medicinal substances

E980.3 Poisoning by tranquilizers and other psychotropic agents, undetermined whether accidentally or purposely inflicted

**Remission or codes not specific to IDU (Use may be oral, rectal, or smoked)**

F15.21 Other stimulant dependence, in remission

T43.621A Poisoning by amphetamines, accidental (unintentional), initial encounter

T43.622A Poisoning by amphetamines, intentional self-harm, initial encounter

T43.623A Poisoning by amphetamines, assault, initial encounter

T43.624A Poisoning by amphetamines, undetermined, initial encounter

**Remission**

305.43 Sedative, hypnotic or anxiolytic abuse, in remission

**Remission or codes not specific to IDU (Use may be oral, rectal, or smoked)**

304.83 Combinations of drug dependence excluding opioid type drug, in remission

292.85 Drug induced sleep disorders

304.63 Other specified drug dependence, in remission

304.93 Unspecified drug dependence, in remission

305.93 Other, mixed, or unspecified drug abuse, in remission

648.31 Drug dependence of mother, delivered, with or without mention of antepartum condition

648.32 Drug dependence of mother, delivered, with mention of postpartum complication

648.34 Drug dependence of mother, postpartum condition or complication

**Non-injection or mother's injection drug use impact on fetus**

F18.10 Inhalant abuse, uncomplicated;

O99.321 Drug use complicating pregnancy, first trimester

O99.322 Drug use complicating pregnancy, second trimester

O99.323 Drug use complicating pregnancy, third trimester

O99.325 Drug use complicating the puerperium

**Injection drug use relates to mother rather than fetus**

771.83 Bacteremia of newborn

**Not specific to IDU**

730.3 Periostitis without mention of osteomyelitis

730.7 Osteopathy resulting from poliomyelitis

730.8 Other infections involving bone in diseases classified elsewhere

722.9 is not specific enough to be the ICD9 correlate to M46.4, and

discitis seems to be captured under 730.2* and M46.2 and M46.3

**Not specific to IDU**

567.22 Peritoneal abscess

567.38 Other retroperitoneal abscess

569.5 Abscess of intestine

572.0 Abscess of liver

590.1 Acute pyelonephritis

709.8 Other specified disorders of skin

709.3 Degenerative skin disorders -- probably too general

709.9 Unspecified disorder of skin and subcutaneous tissue

**Not abscesses or disorders specific to IDU**

K65.1 Peritoneal abscess

K68.19 Other retroperitoneal abscess

K63.0 Abscess of intestine

K75.0 Abscess of liver

N10 Acute tubulo-interstitial nephritis

L94.2 Calcinosis cutis

L98.8 Other specified disorders of the skin and subcutaneous tissue

L02.419 Cutaneous abscess of limb unspecified

L02.91 Cutaneous abscess, unspecified
